# Vasculotide restores the blood-brain barrier after focused ultrasound-induced permeability in a mouse model of Alzheimer's disease

**DOI:** 10.7150/ijms.36775

**Published:** 2021-01-01

**Authors:** Madelaine Lynch, Stefan Heinen, Kelly Markham-Coultes, Meaghan O'Reilly, Paul Van Slyke, Daniel J. Dumont, Kullervo Hynynen, Isabelle Aubert

**Affiliations:** 1 Biological Sciences, Sunnybrook Research Institute, 2075 Bayview Ave. Toronto, ON, Canada M4N 3M5.; 2 Laboratory Medicine & Pathobiology, University of Toronto, 27 King's College Circle, Toronto, ON, Canada, M5S 1A1.; 3 Physical Sciences, Sunnybrook Research Institute, 2075 Bayview Ave. Toronto, ON, Canada M4N 3M5.; 4 Medical Biophysics, University of Toronto, 101 College Street, Toronto, ON, Canada, M5G 1L7.; 5 Vasomune Therapeutics, 661 University Ave #465, Toronto, ON M5G 1M1.

**Keywords:** Vasculotide, blood-brain barrier, transcranial focused ultrasound, Alzheimer's disease

## Abstract

Focused ultrasound (FUS) is used to locally and transiently induce blood-brain barrier (BBB) permeability, allowing targeted drug delivery to the brain. The purpose of the current study is to evaluate the potential of Vasculotide to accelerate the recovery of the BBB following FUS disruption in the TgCRND8 mouse model of amyloidosis, characteristic of Alzheimer's disease (AD). Accelerating the restoration of the BBB post-FUS would represent an additional safety procedure, which could be beneficial for clinical applications.

**Methods:** TgCRND8 mice and their non-transgenic littermates were treated with Vasculotide (250 ng, intraperitoneal) every 48 hours for 3 months. BBB permeability was induced using FUS, in presence of intravenously injected microbubbles, in TgCRND8 and non-transgenic mice, and confirmed at time 0 by MRI enhancement using the contrast agent gadolinium. BBB closure was assessed at 6, 12 and 20 hours by MRI. In a separate cohort of animals, BBB closure was assessed at 24-hours post-FUS using Evans blue injected intravenously and followed by histological evaluation.

**Results:** Chronic Vasculotide administration significantly reduces the ultra-harmonic threshold required for FUS-induced BBB permeability in the TgCRND8 mice. In addition, Vasculotide treatment led to a faster restoration of the BBB following FUS in TgCRND8 mice. BBB closure after FUS is not significantly different between TgCRND8 and non-transgenic mice. BBB permeability was assessed by gadolinium up to 20-hours post-FUS, demonstrating 87% closure in Vasculotide treated TgCRND8 mice, as opposed to 52% in PBS treated TgCRND8 mice, 58% in PBS treated non-transgenic mice, and 74% in Vasculotide treated non-transgenic mice. In both TgCRND8 mice and non-transgenic littermates the BBB was impermeable to Evans blue dye at 24-hours post-FUS.

**Conclusion:** Vasculotide reduces the pressure required for microbubble ultra-harmonic onset for FUS-induced BBB permeability and it accelerates BBB restoration in a mouse model of amyloidosis, suggesting its potential clinical utility to promote vascular health, plasticity and repair in AD.

## Introduction

Transcranial focused ultrasound (FUS) with microbubbles is a minimally invasive technique used to transiently increase the permeability of the blood-brain barrier (BBB) 1. FUS can destabilize the BBB at the level of tight junction proteins, which gradually return to baseline levels 4 hours post-FUS 2,3. To date, the safety profile of FUS has been established in healthy animals, including mice 4, rats 2, rabbits 1 and rhesus macaques 5,6, while the primary clinical use of FUS would be in disease states, in which the BBB may have a compromised restorative capacity. For example, Alzheimer's disease (AD) has significant cerebrovascular pathology, including cerebral amyloid angiopathy (CAA) 7,8, raising questions around whether the plasticity of the BBB and its capacity for repair in response to FUS is compromised 8,9.

FUS alone, and in combination with therapeutics has highlighted its potential in the treatment of AD 10-14. A recent safety clinical trial conducted in a small group of AD patients suggests that functioning of the BBB is restored within 24 hours following FUS 15. To further improve safety and possibly treatment efficacy, promoting a healthy vasculature prior to the administration of FUS may have significant benefits. This could avoid the potential prolonged exposure of the brain to blood components, which could be detrimental. The ability to promote a rapid restoration of the BBB after FUS may significantly contribute to better treatment options for AD patients in the future. As such, we explored the potential of Vasculotide to promote BBB restoration after FUS.

Vasculotide is a 14 kDa angiopoietin-1 (Ang-1) peptide mimetic composed of 4 short synthetic peptides (CHHHRHSF) engineered on a tetrameric polyethylene glycol (PEG) backbone 16,17. The impact of Vasculotide on the brain vasculature has been understudied. Recent data in a model of diabetic stroke demonstrates that Vasculotide has neuroprotective properties including protection from BBB breakdown and significant reductions in markers of neuroinflammation 18. These improvements correlated with increased functioning when assessed by blinded observer for neurological severity score (NSS) 18. In organs other than the brain, Vasculotide was found to decrease vascular endothelial cell permeability in sepsis, endotoxemia, ionizing radiation damage, tumor cell extravasation, influenza, acute kidney injury, dermatitis, pneumonia and hemorrhagic shock 16,17,19-26.

Here, we first compared the closure of the BBB following FUS-induced permeability in a transgenic mouse model of AD (TgCRDN8) with characteristic amyloid pathology 27 to the BBB response in healthy, non-transgenic littermates. We then assessed the ability of Vasculotide to improve the restoration of the BBB following FUS-induced permeability in TgCRND8 and non-transgenic littermates.

Our findings support the potential of Vasculotide to reduce the time required to reseal the BBB following FUS treatments; a therapeutic approach which is being evaluated in several neurodegenerative disorders including AD and amyotrophic lateral sclerosis (ALS) 15,28,29. Our study can have broader implication for Vasculotide as a therapeutic of interest in disease states presenting with a compromised BBB, including stroke and vascular dementia.

## Materials and Methods

### Vasculotide

Drs. Daniel Dumont and Paul Van Slyke at Sunnybrook Research Institute generously provided Vasculotide. Vasculotide was prepared as described by David *et al*. 22. Briefly, a modified T7 peptide (CHHHRHSF) was covalently linked to a four-armed polyethylene glycol (PEG)-maleimide backbone to generate the compound, Vasculotide. Vasculotide was reconstituted in phosphate buffered saline at a concentration of 1 mg/ml and diluted to a working concentration of 2.5 ng/ul.

### Animal Care and Husbandry

Transgenic mice from the Centre for Research in Neurodegenerative Disease (TgCRND8) and their non-transgenic littermates 27 were bred and housed at Sunnybrook Research Institute. TgCRND8 mice are on a mixed C57/C3H background and have a double mutation in the amyloid precursor protein 695 (KM670/671NL, V717F) 27. These mice present with amyloid pathology and cognitive deficits by 3 months of age 27. TgCRND8 mice develop CAA, detectable at 4 months of age and increasingly evident by 5-6 months of age 7.

### Treatment

Three-month-old male and female mice were injected with Vasculotide (250 ng, intraperitoneal), or PBS control every 48 hours starting at 3 months of age (n=6 per group; non-Tg PBS, non-Tg VT, TgCRND8 PBS, TgCRND8 VT). Treatment paradigm was chosen based on CAA pathology 7. Treatment started at 3 months (pre-CAA) and ended at 5-6 months, when CAA is established 7, after which mice underwent MRIgFUS (Table 1). For MRIgFUS, mice were anesthetized with isofluorane, depilated and fitted with a tail vein catheter. Mice were placed supine on an MRIgFUS compatible positioning system fitted for the ultrasound transducer and the 7T MRI (BioSpec 70/30 USR, Bruker, Billerica, Mass). Mice were deeply anesthetized using a cocktail of 15% ketamine and 5% xylazine, perfused transcardially with 0.9% saline and sacrificed 24 hours after MRIgFUS. All animal procedures were conducted with the approval of the Animal Care Committee of Sunnybrook Research Institute and in compliance with the guidelines established by the Canadian Council on Animal Care and the Animals for Research Act of Ontario.

### MR Imaging

T2-weighted MR imaging was used for targeting of FUS to four spots on the left hemisphere. Contrast-enhanced (0.1 ml/kg, Omniscan, GE) T1-weighted images (TR/TE = 500/10 msec) were used to assess BBB permeability immediately after FUS, as described in 30, as well as at 6, 12 and 20 hours post-FUS. Contrast was given as a bolus intravenous injection immediately after FUS. At 6, 12 and 20 hours post-FUS contrast was given as an intraperitoneal injection (0.5 ml/kg) 20 minutes prior to image acquisition, and MR imaging was performed for 15 minutes to ensure enhancement peak was detected.

### Focused Ultrasound

**Figure 1:**
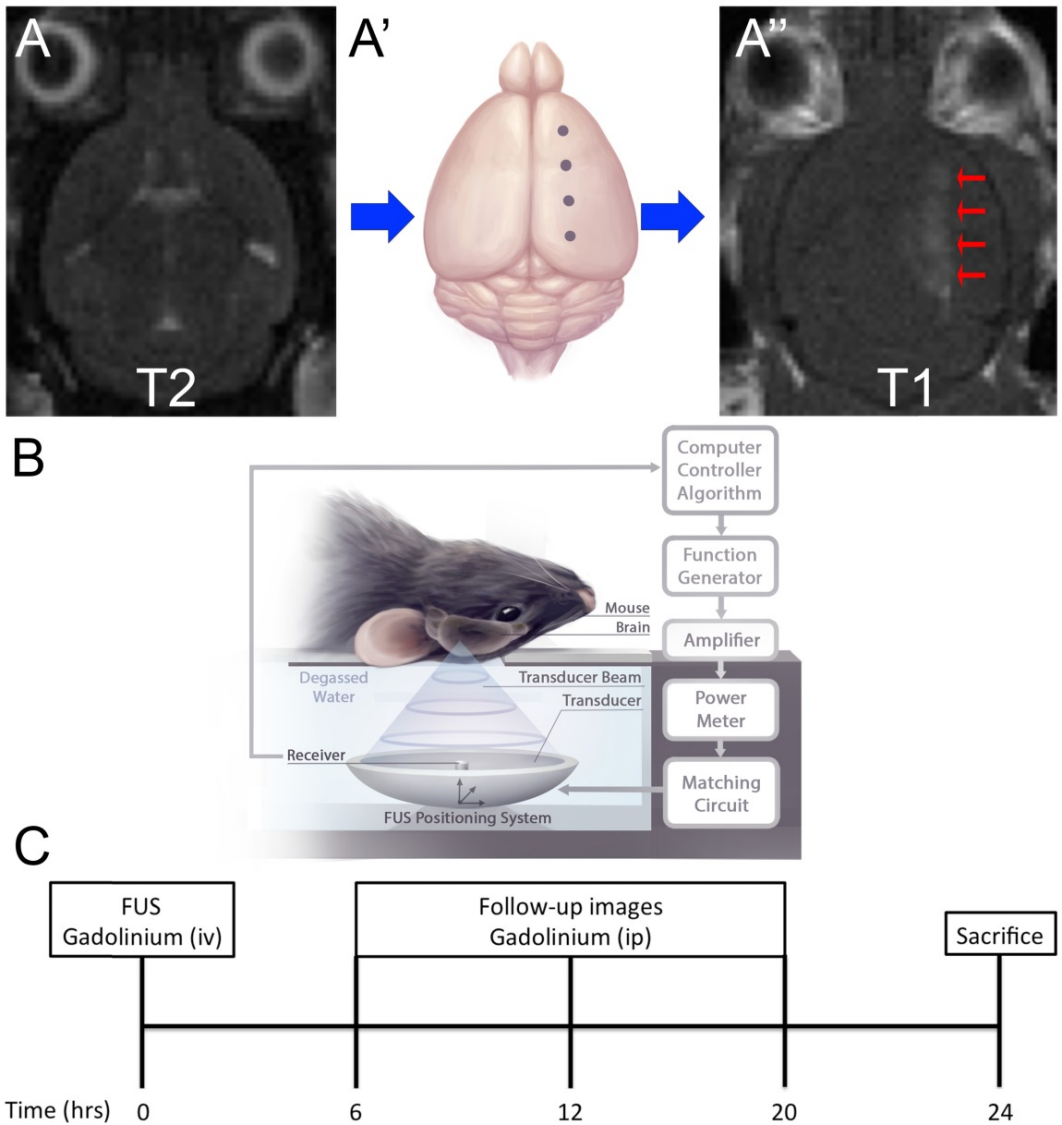
** Experimental Setup and Schematic of Treatment.** *(A) Magnetic Resonance Imaging (MRI) targeting.* Mice were placed in a supine position on an MRI-compatible sled. (A) T2-weighted images were acquired for targeting of FUS beam to four spots along one hemisphere (A'). (A'', arrows) Blood-brain barrier (BBB) permeability was confirmed by gadolinium enhancement using T1-weighted images. *(B) Schematic of FUS treatment.* Mice remained on the MRI-compatible sled and were placed above the transducer for focused ultrasound (FUS) treatment. Briefly, FUS is generated from a transducer, immersed in a water bath, positioned under the mouse. Microbubble emissions are detected by a receiver, which feeds back to a computer controller algorithm to modulate FUS power. *(C) Experimental timeline.* FUS treatment occurred at 0-hour and BBB permeability was confirmed by MRI using an intravenous injection of gadolinium contrast agent. Subsequently images were acquired 6, 12 and 20 hours after FUS using intraperitoneal injections of gadolinium contrast agent. Mice were then sacrificed at 24-hours.

**Figure 2:**
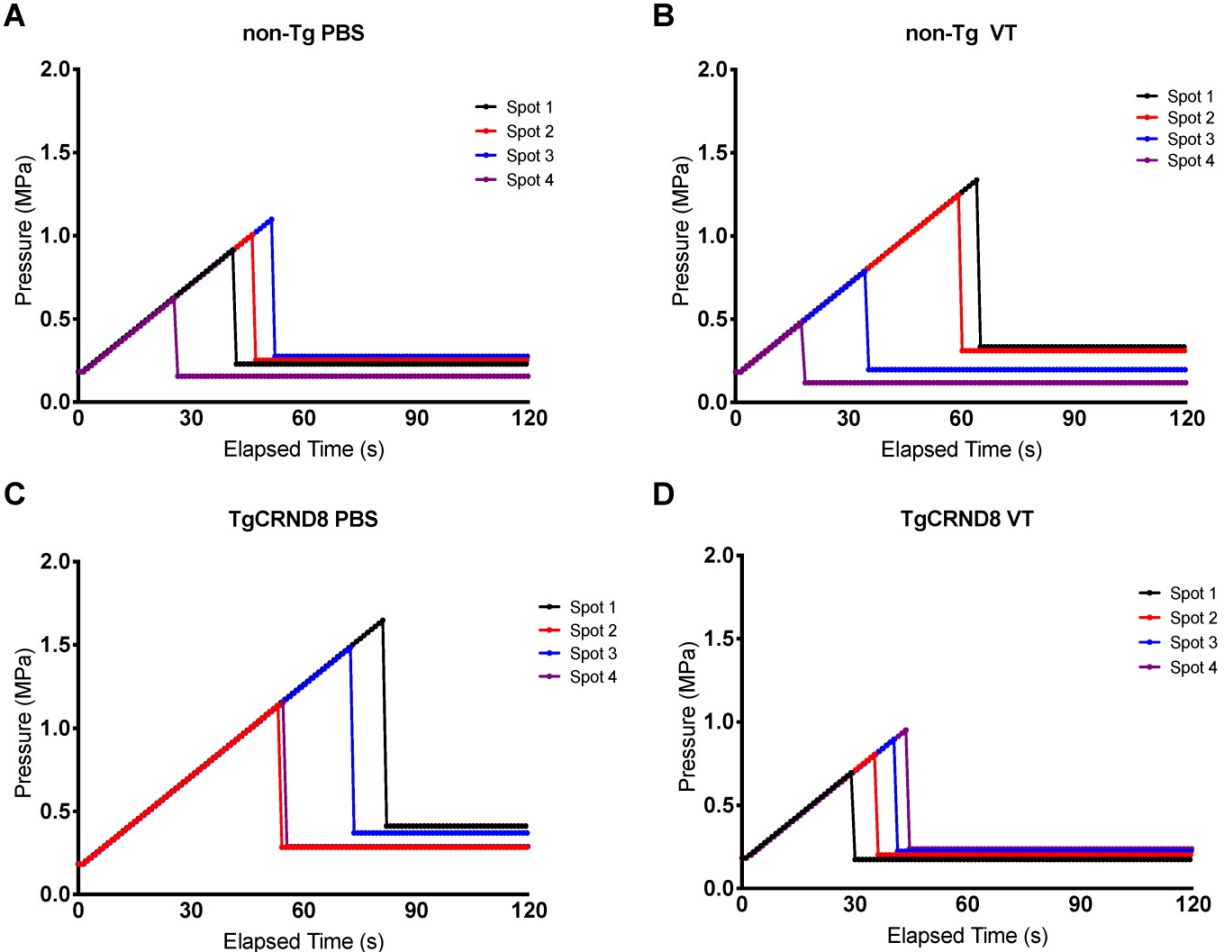
** Pressure drop to 25% of peak pressure to induce blood-brain barrier permeability.** Focused ultrasound pressure increased in steps of 0.02 MPa every second until reaching peak pressure at which point ultra-harmonic signals were detected. The pressure is subsequently reduced to 25% of the peak pressure and maintained to induce blood-brain barrier permeability. Each spot of the four focal spots were monitored independently thereby generating a unique time and peak pressure signature. Graphs are a representative mouse of each genotype and treatment, i.e. (A) non-Tg PBS (B) non-Tg VT (C) TgCRND8 PBS (D) TgCRND8 VT, demonstrating pressure increase (MPa) on the y-axis and time (seconds) on the x-axis.

Focused ultrasound was generated using an in-house fabricated lead zirconate titanate (PZT) transducer (PZT from DeL Piezo Specialties, LLC, West Pal Beach, Florida, USA), with a 75 mm diameter and 60 mm focal length (focal-number = 0.8). Sonications consisted of 10 ms bursts at a frequency of 1.68 MHz and 1 Hz pulse repetition frequency (PRF) for a total of 2 minutes. Ultrasound was targeted to four focal spots on the left hemisphere, with the contralateral hemisphere serving as an untreated control (Fig. 1A). At the time of sonication, mice received an intravenous injection of 0.02 ml/kg Definity microbubbles, diluted 1:50 in saline (Lantheus Medical Imaging, Billerica, Mass, USA) to induce BBB permeability. A custom-built PVDF hydrophone was used to detect microbubble emissions during ultrasound bursts 31. The burst pressure was modulated based on the appearance of sub-harmonic (0.5*f_0_*) and ultra-harmonic (1.5*f_0_*) signals via a real-time control algorithm (Fig. 1B) 32. Pressure was derated to account for the insertion loss of the skull bone (18%) and attenuation through 2.5mm of brain tissue at 5Np/m/MHz 1,33. The pressure was increased from 0.2 MPa in steps of 0.02 Mpa until ultra-harmonic signals were detected, at which point the pressure was decreased to 25% of the peak pressure reached for the remainder of the sonication. Pressure increases were recorded for each independent focal spot every second (Fig. 2). Each of the four focal spots were monitored independently, thus generating four unique time-pressure signatures. This demonstrates the step-wise pressure increase every second until the detection of local ultra-harmonic frequencies (peak pressure), after which the feedback mechanism triggers the pressure at each focal spot to be reduced to 25% of the peak pressure for the remainder of the sonication.

### Quantification of blood-brain barrier permeability

#### MATLAB Analysis of Blood-Brain Barrier Closure

To assess BBB closure mice received intraperitoneal injections of gadolinium contrast agent, Omniscan (0.5 ml/kg), which circulated 20 minutes prior to image acquisition. For the purpose of this study longitudinal intravenous injections in the mouse tail vein were a technical limitation. A catheter cannot be left in for extended period of time, nor could the tail vein be repeatedly injected within the short time frame. Upon inducing BBB permeability, the initial enhancement and coordinates of each focal spot were recorded. In subsequent follow-up images the same coordinates were used to quantify enhancement (Fig. 1C); BBB closure was defined when there was less than two standard deviations of the enhanced relative to the unenhanced hemisphere. Each focal spot represents a distinct vasculature composition and a unique acoustic emission, as detected by the feedback controller. As such, each focal spot was considered independently. Gadolinium contrast enhancement was quantified in MATLAB (Mathworks, Natick, MA) by finding the mean pixel intensity of a 3×3 voxel region of interest surrounding the point of maximum enhancement.

#### Evans Blue Quantification

24-hours after MRIgFUS, a separate cohort of TgCRND8 (n=4) and non-Tg (n=6) mice received an intravenous injection of 4 ml/kg of 2% Evans blue (EB) dye (Table 1). The dye was allowed to circulate 10 minutes prior to sacrifice. Animals were deeply anesthetized using a cocktail of 15% ketamine and 5% xylazine and perfused transcardially with 0.9% saline followed by 4% paraformaldehyde. Brains were isolated, post-fixed in 4% paraformaldehyde for 24 hours then transferred to 30% sucrose.

The ten 24-hour and one 6-hour, post-FUS brains were cut on a vibratome at 0.5 mm for optical imaging using a Xenogen IVIS 200. One brain was collected immediately after FUS and cut at 1 mm for optical imaging. Fluorescence data was collected using the Cy5.5 filter with an exposure time of 0.5 s. Known quantities of EB were spotted onto filter paper to create a standard curve, allowing quantification of EB from regions of interest within the brain as described by Jaffer et al. 34.

### Immunohistochemistry

Brains for immunohistochemistry were cut axially at a thickness of 40 μm. Free floating sections were washed 3 times in PBS + 0.3% Triton X100, pH 7.4 prior to incubation in blocking solution (PBS, 3% BSA, 0.3% Triton X100, 3% donkey serum) for 3 hours at room temperature.

Primary and secondary antibodies were incubated in blocking solution overnight at 4°C. Fibrinogen was stained using the polyclonal anti-human fibrinogen antibody (Dako, A0080, 1:100). Erythrocytes were stained using the rat anti-mouse Ter119 antibody (BD Pharmingen, 553671, 1:100). Mouse IgG was stained and detected using the donkey anti-mouse IgG-Cy5 antibody (Jackson ImmunoResearch, 715-175-150, 1:200). Staining for fibrinogen and erythrocytes was detected using donkey anti-rabbit-Cy3 antibody (Jackson ImmunoResearch, 711-162-152, 1:500) and donkey anti-rat 488 (Jackson ImmunoResearch, 712-545-150, 1:500), respectively. Whole sections were imaged on the TissueScope 4000 fluorescence tissue scanner (Huron Digital Pathology) at a scan resolution of 0.5 μm/pixel and constant PMT settings.

### Statistical Analysis

All statistical analysis was done using GraphPad Prism. Enhancement and BBB disruption pressure were analyzed using a 2-way ANOVA with a Tukey post-hoc test. Restoration of the BBB was analyzed by log-rank (Mantel-Cox). Multiple comparisons amongst BBB restoration curves were done individually (4 comparisons), and corrected for multiple comparisons using the Bonferroni corrected threshold; the significance level of 0.05 was divided by the number of comparisons (4) resulting in a multiple comparisons significance threshold of 0.0125. Multiple comparisons in which *p*<0.0125 were considered statistically different. Significance is defined as: *p<0.05, **p<0.01, ***p<0.001.

## Results

### The blood-brain barrier is impermeable to Evans blue 24-hours post-FUS

**Figure 3:**
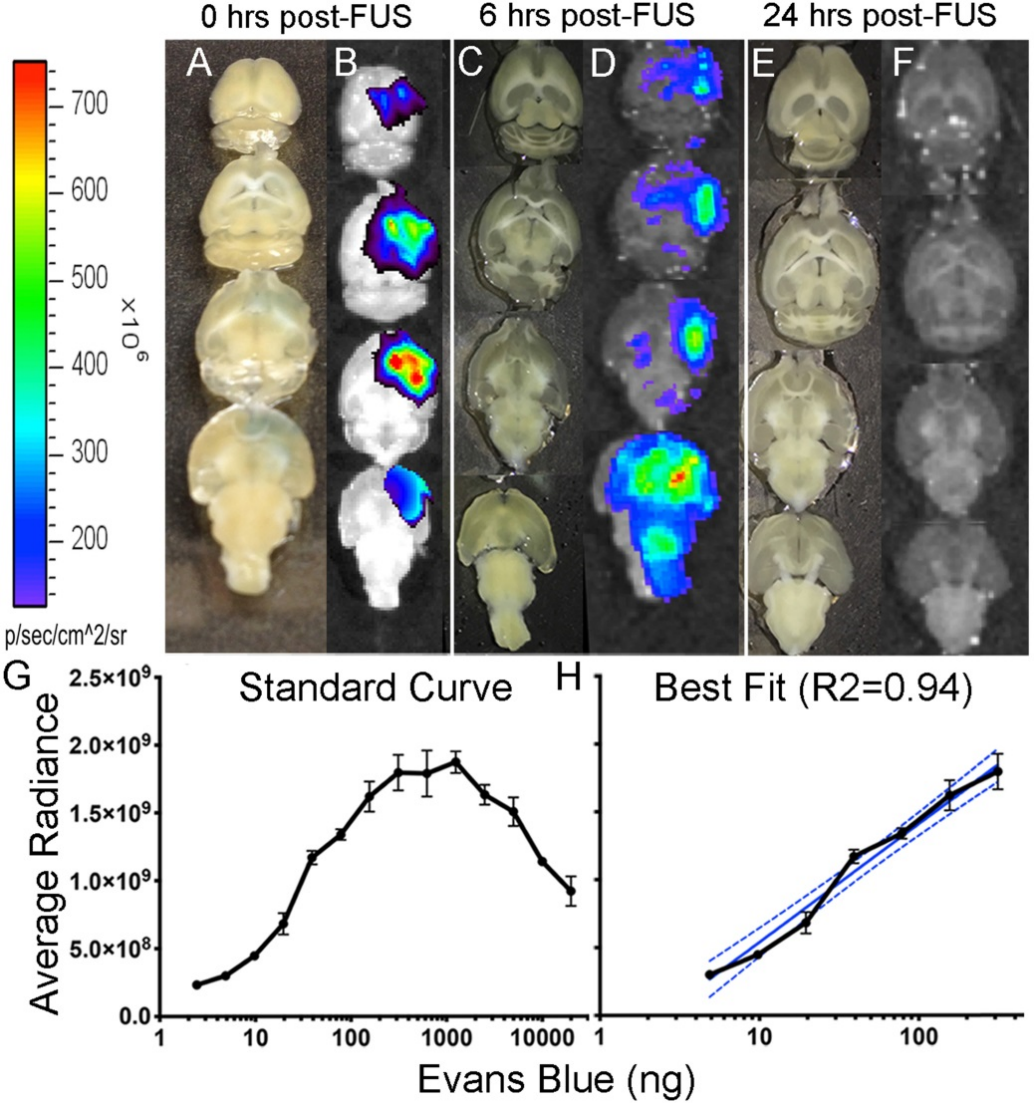
** Detection of Evans blue after focused ultrasound-induced blood-brain barrier permeability.** Images of Evans blue dye in brain cut at 1 mm, 0-hours after FUS (A), in which 162 ng of Evans blue were detected by optical imaging (B); Images of brain cut at 500 µm, 6-hours after FUS (C), in which 33 ng of Evans blue were detected by optical imaging; Images of brains cut at 500 µm, 24-hours after FUS (E) in which no Evans blue was detected by optical imaging in both non-Tg (shown here) and TgCRND8 mice (F). Standard curve was created by fluorescent detection of known quantities (ng) of Evans blue using a Xenogen IVIS 200 Optical Imaging System. The quantity of Evans blue was plotted logarithmically relative to the average radiance (a measure of photons/pixel) (G). A semi-log line of best fit (R^2^ = 0.94) was fitted to the linear portion of curve “C” and used to interpolate Evans blue quantities (H). *Colour scale was generated in relation to the images in panel “D”.*

In our experimental design gadolinium was injected intraperitoneally to detect contrast in the brain following FUS. This led to prolonged detection of BBB permeability, as evidenced by gadolinium detection at the 20-hour time point (Fig. 3). While this is within the range of closure reported in other studies 35,36, most investigators have used intravenous gadolinium 30, Evans blue 2 and other imaging metrics such as electron microscopy 37 to define BBB closure. Here, we sought to corroborate these previous closure findings, independently of Vasculotide. This was done in a separate cohort of mice by EB intravenous injection (2%, 4 ml/kg) at 6 and 24-hrs post-FUS, brain isolation and quantitative EB fluorescent detection (Fig. 3D, F).

Known quantities of EB were used to generate the logarithmic standard curve (Fig. 3G). From this curve, a semi-log line was fitted to the linear portion (R^2^ = 0.94; Fig. 3H) to interpolate the quantities (ng) of EB. Quantification of EB immediately after FUS revealed 162 ng of EB (Fig. 3B) in the brain and only 33 ng of EB 6-hours after FUS (Fig. 3D). EB injections 24-hours after FUS did not show any EB visible by eye or detected by fluorescence in TgCRND8 or non-Tg mice (Fig. 3F). The lack of EB post-FUS indicates that the BBB is impermeable at 24-hours in both non-Tg and TgCRND8 mice.

### Vasculotide reduces the ultra-harmonic threshold required for blood-brain barrier disruption in TgCRND8 mice

Our method for the application of FUS uses a control algorithm to safely modulate the pressure required to induce BBB permeability. Microbubble acoustic emissions are detected after each burst, which facilitates the stepwise increase in applied pressure. Detection of sub-harmonic or ultra-harmonic emission is regarded as the upper safety limit, after which the program reduces the applied pressure to the predetermined level of 25% to induce BBB permeability 32.

**Figure 4:**
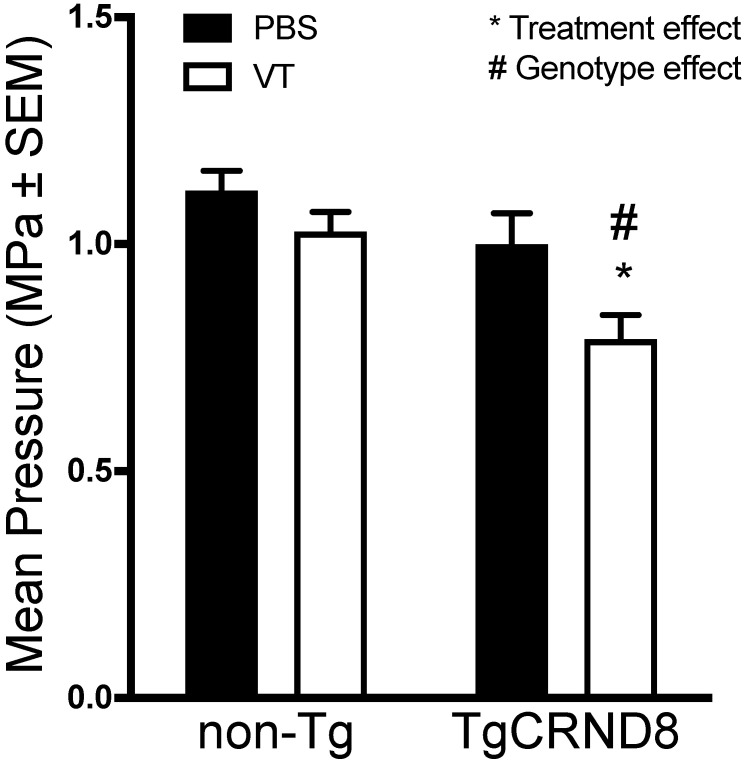
** Vasculotide reduces the threshold for sub- and ultra-harmonic bubble behaviour in TgCRND8 mice.** Focused ultrasound transducer detects sub-harmonic frequencies of injected microbubbles, gradually increasing pressure to induce blood-brain barrier (BBB) permeability until sub-harmonic emissions are detected at which point the pressure is reduced to 25% and maintained to induce BBB permeability. Maximum pressures were recorded for each focal spot. Data presented as mean+SEM, N=24 focal spots per group. Statistical analysis was done using a 2-way ANOVA with a Tukey's multiple comparisons post-test. # genotype effect * treatment effect as calculated by Tukey's multiple comparisons.

Analysis of the maximum pressure reached by the ultrasound transducer demonstrated no statistical difference in the maximum threshold required to induce sub- and ultra-harmonic bubble behaviour between TgCRND8 and non-Tg mice. Vasculotide treatment had no effect on the threshold required in non-Tg mice. However, TgCRND8 mice treated with Vasculotide (TgCRND8-VT) had a 21-29% lower threshold to induce sub- and ultra-harmonic bubble behaviour when compared to all other groups (Fig. 4, TgCRND8-VT compared to TgCRND8-PBS *p=*0.03; non-Tg-VT, *p=*0.01; non-Tg-PBS, *p=*0.0002). A Grubbs test was conducted and demonstrated no significant outliers.

### Vasculotide does not impact the initial enhancement in TgCRND8 mice

**Figure 5:**
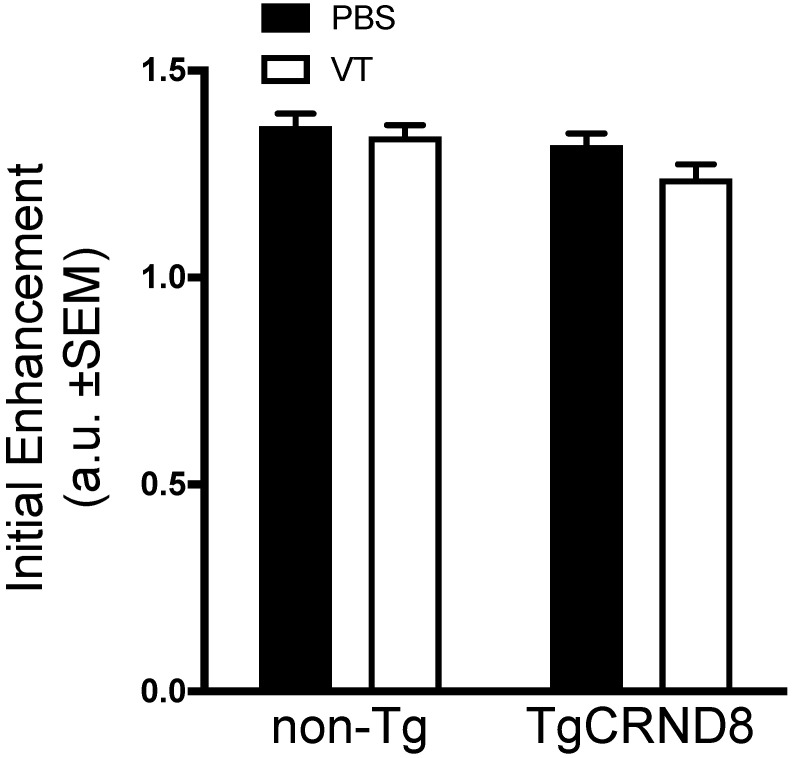
** Vasculotide does not impact the initial enhancement after focused ultrasound in TgCRND8 mice.** Gadolinium enhancement immediately after focused ultrasound -mediated blood-brain barrier permeability was quantified by the intensity of a 3x3 voxel region using a custom program in MATLAB. Data presented as the ratio of gadolinium enhancement between the enhanced and unenhanced hemispheres as mean + SEM, N=21-24 focal spots per group. Statistical analysis was done using a 2-way ANOVA with a Tukey multiple comparisons post-test.

Immediately after FUS sonication, mice received an intravenous dose of gadolinium to confirm BBB permeability. Quantification of gadolinium contrast revealed no significant difference in initial enhancement between TgCRND8 and non-Tg mice. Analysis of Vasculotide treated mice did not demonstrate any significant enhancement differences in either non-Tg or TgCRND8 mice (Fig. 5).

### Vasculotide accelerates the closure of the blood-brain barrier post-FUS

**Figure 6:**
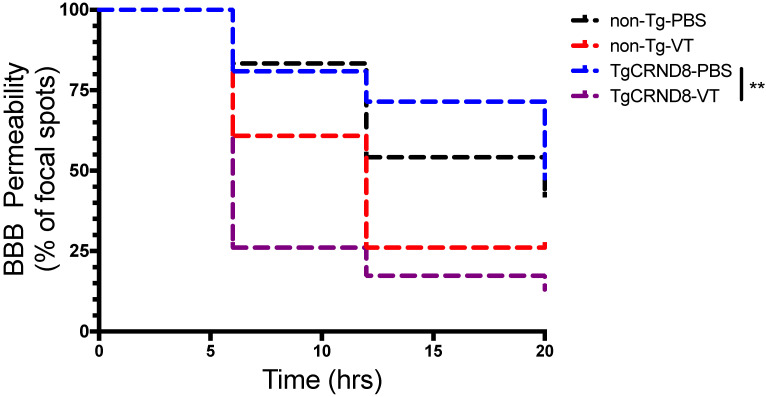
** Vasculotide promotes blood-brain barrier restoration in TgCRND8 mice after focused ultrasound-induced permeability.** Results are expressed as the percentage of focal spots permeable to gadolinium at 6, 12 and 20 hours after focused ultrasound (i.e. at time 0, 100% of focal spots are permeable). Closure was defined as less than two standard deviations between the enhanced and unenhanced hemisphere. Statistical analysis was done using a log rank (Mantel-Cox) test. Multiple comparisons were calculated at a Bonferroni corrected threshold of 0.0125 to determine significance. N = 21-24 focal spots per line, **p<0.01, ***p<0.001.

In TgCRND8 mice, Vasculotide treatment significantly reduced BBB closure time post-FUS, as indicated by the fewer number of focal spots permeable to gadolinium within the 20-hour time frame (Fig. 6, solid black line TgCRND8-VT). Specifically, the restoration of the BBB in Vasculotide treated TgCRND8 mice is indicated by the drop in the number of foci where gadolinium was detected at time zero (100%) of FUS-induced BBB opening to 26%, 17% and 13% of foci remained permeable to gadolinium at 6, 12 and 20 hours, respectively (Fig. 6, black solid line). In Vasculotide treated non-Tg mice, approximately 61%, 26% and 26% of foci remained permeable to gadolinium at 6, 12 and 20 hours, respectively (Fig. 6, black dashed line). Log-rank analysis revealed a statistically significant difference between TgCRND8-VT and TgCRND8-PBS (*p=*0.001).

Comparison of non-Tg and TgCRND8 mice revealed no significant difference in gadolinium permeability with respect to genotype (Fig. 6). In non-Tg mice, 83%, 54% and 42% of foci remained permeable to gadolinium at 6, 12 and 20 hours, respectively (Fig. 6, red dashed line). In TgCRND8 mice, 81%, 71% and 48% of foci remained permeable to gadolinium at 6, 12 and 20 hours, respectively (Fig. 6, red solid line).

Taken together these results indicate a substantial effect of Vasculotide in accelerating the closure of the BBB after FUS. The total number of spots closed by 20 hours post-FUS was substantially greater in Vasculotide treated TgCRND8 mice with 86% of foci closed compared to PBS treated TgCRND8 mice in which 52% of foci were closed (Fig. 6, black compared to red solid lines).

### Extravasation of blood-borne components is consistent with prolonged gadolinium enhancement

**Figure 7:**
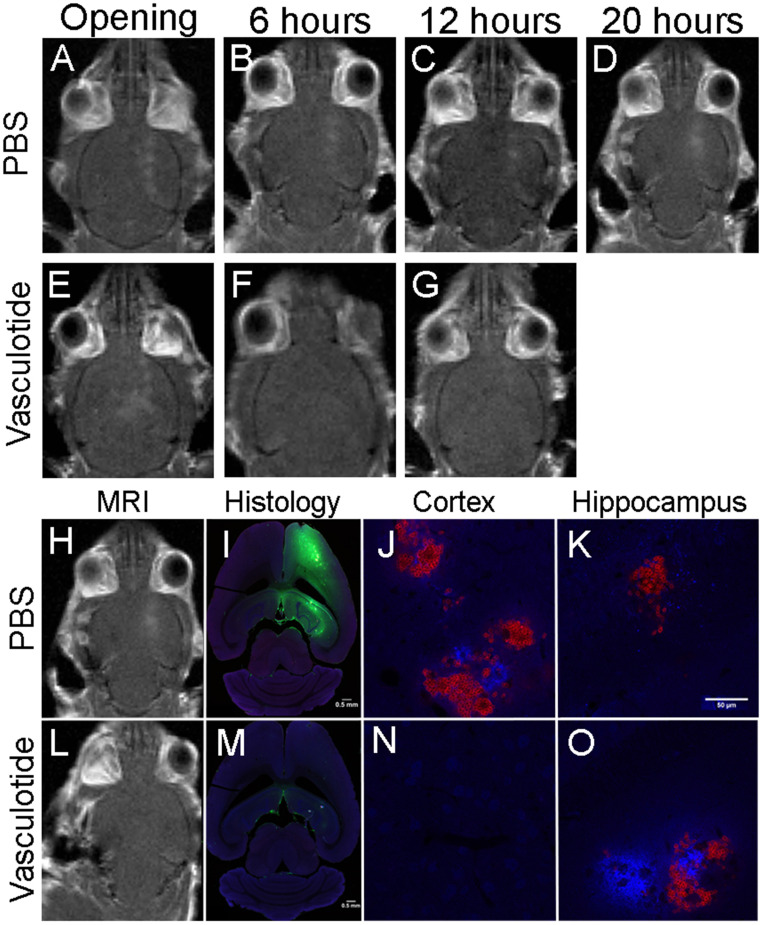
** Gadolinium enhancement is consistent with blood-borne components extravasation at 24-hours.** Representative images of PBS and Vasculotide treated TgCRND8 mice, which were defined (through gadolinium enhancement) as complete closure with Vasculotide and incomplete closure with PBS. Gadolinium enhancement in PBS treated TgCRND8 mouse at (A) opening, (B) 6-hours, (C) 12-hours and (D) 20-hours. Gadolinium enhancement in Vasculotide treated TgCRND8 mouse at (E) opening, (F) 6-hours and (G) 12-hours. (I, M) Histology sections 24-hours after FUS demonstrate IgG (green) in locations consistent with gadolinium enhancement (H, L). Extravasation of fibrinogen (blue) and erythrocytes (red) are demonstrated in the 20X magnification images in the cortex (J, N) and hippocampus (K,O). MR images were reflected right/left to match histology images.

Gadolinium enhancement illustrated focal spots where BBB permeability was induced up to 20-hours post-FUS. Histological analysis of IgG (green), fibrinogen (blue) and erythrocyte (red) extravasation 24-hours post-FUS coincided with the locations of gadolinium permeability (Fig. 7). The extent of extravasation appears consistent with the rate of BBB closure. Qualitatively, mice that demonstrated rapid BBB restoration, such as the Vasculotide treated TgCRND8 mice (Fig. 7), had reduced entry of blood-borne molecules as compared to the PBS treated TgCRND8 mice (Fig. 7), which remained permeable to gadolinium.

## Discussion

The ability of FUS to permeabilize the BBB for the delivery of therapeutics to the brain offers exciting new possibilities for the treatment of AD and other neurological diseases. Yet, important questions and potential concerns remain to be considered, such as how long the BBB remains permeable in presence of amyloid pathology and to what blood-borne molecules the brain will be exposed? As such, we investigated the timeline of the BBB closure after FUS in a mouse model with amyloid pathology, and whether BBB restoration could be accelerated with Vasculotide. Following FUS, BBB opening parameters, namely the pressure required for ultra-harmonic microbubble emissions, was reduced in Vasculotide-treated TgCRND8 mice. Additionally, Vasculotide treatment significantly accelerated BBB closure in TgCRND8 mice. Neither FUS-related BBB opening parameters nor closure differed between TgCRND8 and non-transgenic mice. While the presence of amyloid pathology alone does not alter FUS-induced BBB permeability, the results shown here indicate that the combination of amyloid pathology and Vasculotide treatment can influence FUS-induced BBB permeability and closure.

The presence of CAA in the TgCRND8 mice has been demonstrated as early as 4 months of age, causing changes in vascular morphology and functional deficits 7,38. Previous research using FUS to open the BBB, without the use of the feedback controller 32, required twice the dosage of microbubbles in TgCRND8 mice (80 ul/kg) as compared to non-transgenic littermates (40 ul/kg) in order to induce BBB permeability 11. The requirement of twice the microbubble dosage at a constant pressure for FUS-induced BBB permeability, suggests an altered vascular response to FUS in TgCRND8 mice. Furthermore, after FUS, Burgess *et al*. showed a reduced permeability constant in TgCRND8 mice and an increase of slow leaking vessels attributed to the presence of CAA 39. Despite differences in leakage kinetics, the acoustic pressure and probability of inducing BBB permeability remained the same between genotypes 39. Therefore, in TgCRND8 mice although leakage occurs slower in response to FUS, the capacity for inducing BBB permeability is comparable to that of non-Tg mice 39. Consistent with the study by Burgess *et al.* the results presented here show no disparities in acoustic pressure and permeability to gadolinium up to 20-hours after FUS between genotypes. This suggests that the TgCRND8 mice possess a similar capacity for repair as the non-Tg mice, facilitating safe application of FUS. Interestingly, Vasculotide reduced the upper ultra-harmonic threshold required to disrupt the BBB in the TgCRND8 mice. This suggests that the effect of Vasculotide in the TgCRND8 mice may alter the response of the vasculature to ultrasound exposure. Vasculotide treatment resulted in detection of sub- and ultra-harmonic emissions at lower pressures, suggesting that less pressure may be required to induce BBB permeability. Although the exact mechanism of Vasculotide in the brain has yet to be determined, it is possible that Vasculotide treatment improves vascular plasticity thereby making the cerebrovasculature more amenable to FUS-induced BBB permeability. Having shown a reduction in the ultra-harmonic threshold with no change in initial enhancement indicates that Vasculotide treatment can reduce the acoustic threshold required for BBB permeability without impeding the opening volume for potential therapeutic delivery.

Furthermore, Vasculotide treatment has a marked effect on reducing BBB permeability after FUS. In TgCRND8 mice chronic Vasculotide treatment not only increased the total number of focal spots impermeable to gadolinium within the 20-hour time frame, but also produced a more rapid restoration of the BBB. Previous studies have demonstrated the ability of acute Vasculotide treatment to improve endothelial barrier function and reduce vascular leakage in the lung and kidney 17,40. Vasculotide has been shown to increase VE-cadherin, PECAM-1 and Sma1, likely contributing to the endothelial barrier improvement 16,22. Therefore, it is plausible that Vasculotide produces a similar effect here in reducing brain endothelial cell permeability, possibly by increasing junction proteins or altering their cellular localization.

The results presented here suggest prolonged permeability to intraperitoneal gadolinium as long as 20-hours post-FUS. This permeability appears to be independent of genotype, as both non-Tg and TgCRND8 mice demonstrated extended gadolinium permeability. Research has shown that measurements evaluating the time it takes to restore the BBB after FUS is largely dependent on the molecular size of the tracer agent and FUS sonication parameters. Indeed, studies using different molecular size markers of BBB permeability, such EB (~70 kDa), have cited BBB closure occurring 4-6 hours after FUS, with confirmation of closure at 24 hours, 72 hours and 4 weeks later 1,2,30,37,41. Using MR contrast agents of various sizes (1-65 nm) Marty et al. demonstrated that the BBB is impermeable as rapidly as 90 minutes to the 7 nm P792, while Dotarem (1 nm) crossed the BBB up to 24 hours 36. Using HRP (40 kDa) and lanthanum chloride (139 Da), Sheikov et al. showed closure of the BBB at both 6 and 24 hours 2. However, using various exposure times of 6, 8 and 10 s in rabbits, Mei et al. demonstrated the BBB remained permeable to gadolinium contrast agent as late as 24-hours after FUS 41. Furthermore, Samiotaki et al. used various acoustic pressures (0.30-0.60 MPa) and pulse lengths (0.67 μs - 6.7 ms) to demonstrate BBB permeability to gadolinium up to 72 hours post-FUS 35. Similar to our study, Samiotaki used intraperitoneal injections of gadolinium at higher doses than the intravenous injections, which may allow greater sensitivity to small changes in the BBB 35,42. The finding of BBB permeability at the 20-hour time point is relative to the delivery and size of gadolinium used. Studies from our labs and others 35,42 have supported the use of intraperitoneal gadolinium. However, the kinetics of this injection route differs from intravenous, which may thus be the source of prolonged enhancement. Work by Choi *et al.* 4 used intraperitoneal gadolinium, suggesting a slower time course of entry than intravenous and detected enhancement up to 28-hours after FUS. Therefore, the finding that Vasculotide substantially reduces BBB permeability to intraperitoneal gadolinium, suggests that Vasculotide may have a potent effect on the restoration of vascular stability following FUS.

Additionally, it is important to note that gadolinium is relatively small (574 Da) compared to EB (~70 kDa in circulation), thus larger tracers may produce more rapid closure times and may not reflect the size of biological molecules (e.g. albumin 67-69 kDa, globulins >90 kDa) to which the BBB would be exposed 35. Furthermore, the report by Sheikov et al. not only demonstrated that HRP and lanthanum chloride did not pass beyond the endothelial lining by 6-hours post FUS, but also that tight junctions were reformed after 6 hours 2. This suggests that although the BBB may be permeable to gadolinium, the ability of large, potentially toxic molecules is rapidly diminished after FUS.

Previous studies have not detected overt edema, red blood cell extravasation or tissue injury after FUS 4,5,30,32,35,43. Although Alonso et al. detected albumin in the brain parenchyma beginning 30 minutes post-FUS, this was phagocytized by glial cells and cleared from the brain as early as 1-hour and up to 24-hours post-FUS 44. Similarly, the results presented here indicate extravasation fibrinogen, IgG and erythrocytes 24-hours post-FUS, particularly in the mice with prolonged BBB opening (Fig. 7). However, injection of EB at 24-hours post-FUS did not cross the BBB in either non-Tg or TgCRND8 mice (Fig. 3B), suggesting the aforementioned blood-borne molecules likely entered the parenchyma at an earlier time point. Previous work by Jordão et al*.* in TgCRND8 mice demonstrated elevated levels of endogenous antibodies (IgG, IgM) 4-days after FUS 11. These endogenous antibodies co-localized with amyloid-β plaques and could contribute to FUS-mediated plaque reduction 11,14,43. Taken together, it is conceivable that 24-hours after FUS the BBB is impermeable to the entry of additional blood-borne molecules, while Vasculotide treatment reduced the initial entry and/or subsequent detection of blood-borne molecules.

The results presented here regarding the use of FUS to safely induce BBB permeability in TgCRND8 mice are consistent with previous studies by Jordão et al., and Burgess et al. 11,39,43. Yet, we have demonstrated for the first time the applicability of Vasculotide in reducing BBB closure time after FUS. Our current study employed a chronic Vasculotide treatment, and it is possible that shorter treatment durations may be sufficient. Indeed, Vasculotide has been shown to reduce vascular permeability in peripheral organs using acute paradigms. As examples, treatment with Vasculotide 16- and 1-hour before, and 24 and 48-hours post cecal-ligation-and-puncture was sufficient to protect against vascular leak in sepsis 17. Additionally, Vasculotide treatment 7-hours before a 70% lethal dose of lipopolysaccharide administration protected against endotoxemic lung injury 22. Vasculotide post-treatment has also demonstrated success against ionizing radiation damage 19 and influenza 21. Future research using an acute Vasculotide treatment paradigm may also prove efficacious to restore BBB integrity.

Overall, our data demonstrates that Vasculotide is able to accelerate BBB restoration after FUS in the presence of amyloid pathology in the TgCRND8 mouse model of AD. These results indicate a potential role for Vasculotide as a treatment to promote BBB restoration in response to FUS-induced permeability. Furthermore, the impact of Vasculotide on the BBB could conceivably be extended to cases of stroke-as supported by a recent study from Venkat et al. in a preclinical model of stroke 18, neurodegenerative diseases and vascular disorders where the BBB is compromised.

## Limitations

A limitation of our study is that we were unable to detect changes in the molecular components of the BBB in response amyloid pathology or Vasculotide. Using qRT-PCR we did not find significant differences in the transcript levels of ZO-1, occludin and claudin-5 between TgCRND8 and non-Tg mice (Supp. Fig. 1). Furthermore, treatments with Vasculotide did not influence these BBB-related transcripts 45. Additionally, we found that Tie1 and Tie2 transcript levels did not differ in TgCRND8 mice compared to non-Tg (Supp. Fig. 2A, B) 45. The expression levels of Ang1, however, were higher in TgCRND8 compared to non-Tg mice (Supp. Fig. 2C) 45. With all being considered, we were unable to determine the mechanisms by which Vasculotide acts on the BBB to accelerate its restoration.

Future studies are required to characterize the effect of Vasculotide on the molecular and morphological vessel architecture, which was never done in the brain. At the level of cell junctions, *in vitro* and in mouse lung tissue, Vasculotide has been shown to increase VE-cadherin 22. Vasculotide has also been shown to reduce TNF⍺ in the brain 18, which is known to alter BBB permeability by upregulation of ICAM-1 and VCAM-1 46. Indeed, Vasculotide has also been demonstrated to reduce ICAM-1 and VCAM-1 levels 24,47. Vasculotide has been shown to increase angiogenesis in wound healing assays 16. It is known that TgCRND8 mice have increased arteriolar and capillary tortuosity and impaired vascular reactivity associated with cerebral amyloid angiopathy 7. As such, it is plausible that Vasculotide treatment could alter the vessel architecture and plasticity which can contribute to the vascular response to FUS and modulation. Ultimately, there are still gaps in our understanding of how both FUS and Vasculotide modulate the brain vasculature. It is possible that Vasculotide initiates a cascade of events which ultimately result in the reduction of BBB permeability following FUS.

## Supplementary Material

Supplementary methods and figures.

## Figures and Tables

**Table 1:** Experimental Design

	Mice			Analysis
	Start Age	Treatment Groups (n)	End Age	
Chronic vasculotide (VT)	3 months	non-Tg-VT (6)	5-6 months	Post-FUS: At 6, 12, 20-hrs BBB permeability monitored by MRI (gadolinium); Sacrificed at 24-hrs.
		non-Tg-PBS (6)		
		TgCRND8-VT (6)		
		TgCRND8-PBS (6)		
BBB closure to Evans blue (EB)	7 months	EB 0-hrs post-FUS: non-Tg (1)	7 months	Brain cut at 1 μm; optical EB imaging.
		EB 6-hrs post-FUS: non-Tg (1)		Brain cut at 500 μm; optical EB imaging.
	5 months	EB 24-hrs post-FUS: non-Tg (6); TgCRND8 (4)	5 months	Brains cut at 500 μm; optical EB imaging.

## Abbreviations

FUS: focused ultrasound
BBB: blood-brain barrier
AD: Alzheimer's disease
CAA: cerebral amyloid angiopathy
VT: vasculotide
Ang-1: angiopoietin-1
PEG: polyethylene glycol
EB: Evans blue
