# Supplementary material for: Vasculotide restores the blood-brain barrier after focused ultrasound-induced permeability in a mouse model of Alzheimer's disease
Source: Int J Med Sci. 2021 Jan 1;18(2):482–93. doi: 10.7150/ijms.36775 (PMC7757142; doi:10.7150/ijms.36775)
Supplement: Supplementary file 1 — Supplementary methods and figures. [file ijmsv18p0482s1.pdf]

Blood Vessel Isolation

Whole brains from 6 non-transgenic and 6 TgCRND8 mice were collected for blood vessel isolation. Samples were pooled to increase yield, and analyzed as technical replicates. Blood vessels were isolated as described by Hawkes and McLaurin(1). Briefly, the brains were homogenized (0.1M ammonium carbonate, 5mM EDTA, 0.1% sodium azide, 1% sodium orthovanadate, 1% protease inhibitor cocktail) in a dounce using 6 strokes after which homogenates were centrifuged at 100,000g (1hr, 4°C). The pellet was resuspended in 0.1M ammonium carbonate, 7% SDS, 1% sodium orthovanadate and 2% protease inhibitor cocktail, and stirred on ice for 3 hours. Suspension was then filtered through a 40μm filter to isolate blood vessel tufts.

RNA extraction and gene expression analysis

Brain sections were homogenized in TRIzol using a bead homogenizer (Minilys, Bertin Instruments) and RNA extracted with chloroform. Homogenates were centrifuged at 12,000 x g for 15 minutes at 2°C. The clear supernatant was collected and RNA was isolated using the Pure Link RNA mini Kit (Life Technologies), according to manufacturer's instructions. cDNA was synthesized with the Superscript III Reverse Transcriptase (Invitrogen), according to manufacturer's instructions.

RT-PCR was performed using SYBRGreen, on a Viia7 Real-Time PCR System (Applied Biosystems). The following primers were used: *Tie1*F*:* 5'-GCCCTTTTAGCCTTGGTGT-3', *Tie1*R*:* 5'-TTCACCCGATCCTGACTGGTA-3'; *Tie2*F*:* 5'-TGGAGTCAGCTTGCTCCTTT-3', *Tie2*R*:* 5'-ACCTCCAGTGGATCTTGGTG-3'; *Angpt1*F*:* 5'-GGGGGAGGTTGGACAGTAA-3', *Angpt1*R*:* 5'-CATCAGCTCAATCCTCAGC;

-3’, *Angpt2*F*:* 5’-GATCTTCCTCCAGCCCCTAC-3’, *Angpt2*R*:* 5’-TTTGTGCTGCTGTCTGGTTC-3’; *HPRT*F: 5’-CCAGCAAGCCTTGCAACCTTAACCA-3’, *HPRT*R: 5’-GTAATGATCAGTCAACGGGGGAC-3’; *GAPDH*F: 5’-CGACTTCAACAGCAACTCCCACTCTTCC-3’, *GAPDH*R: 5’-TGGGTGGTCCAGGGTTTCTTACTCCTT-3’

Statistical Analysis

Data is expressed as fold change + 95% confidence interval. Analysis of regional expression within a single age group was done using a 1-way repeated measures ANOVA with a Tukey’s post-hoc test. Analysis of regional expression across age groups or genotype was done using a 2-way repeated measures ANOVA with a Sidak’s post-hoc test. Analysis of individual gene expression by genotype was done by two-tailed Student’s t-test. Statistical analysis was performed using GraphPad Prism 8, $p<0.05$ was considered statistically significant (*$p<0.05$, **$p<0.01$, ***$p<0.001$, ****$p<0.0001$).

References

1. Hawkes CA, McLaurin J. Selective targeting of perivascular macrophages for clearance of β-amyloid in cerebral amyloid angiopathy. Proc Natl Acad Sci USA. National Acad Sciences; 2009 Jan 27;106(4):1261–6.

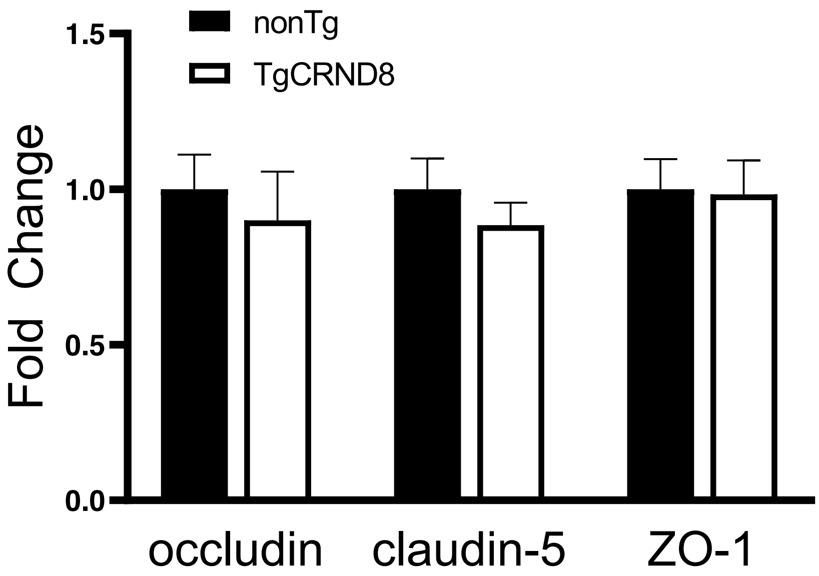

Supp Figure 1: Expression of tight junction proteins are not modulated in blood vessels of TgCRND8 mice. Expression of occludin, claudin-5 and ZO-1 in blood vessels isolated from whole brains of 6-month old TgCRND8 mice. Fold change is relative to non-transgenic mice. Statistical analysis done using a student's unpaired t-test.

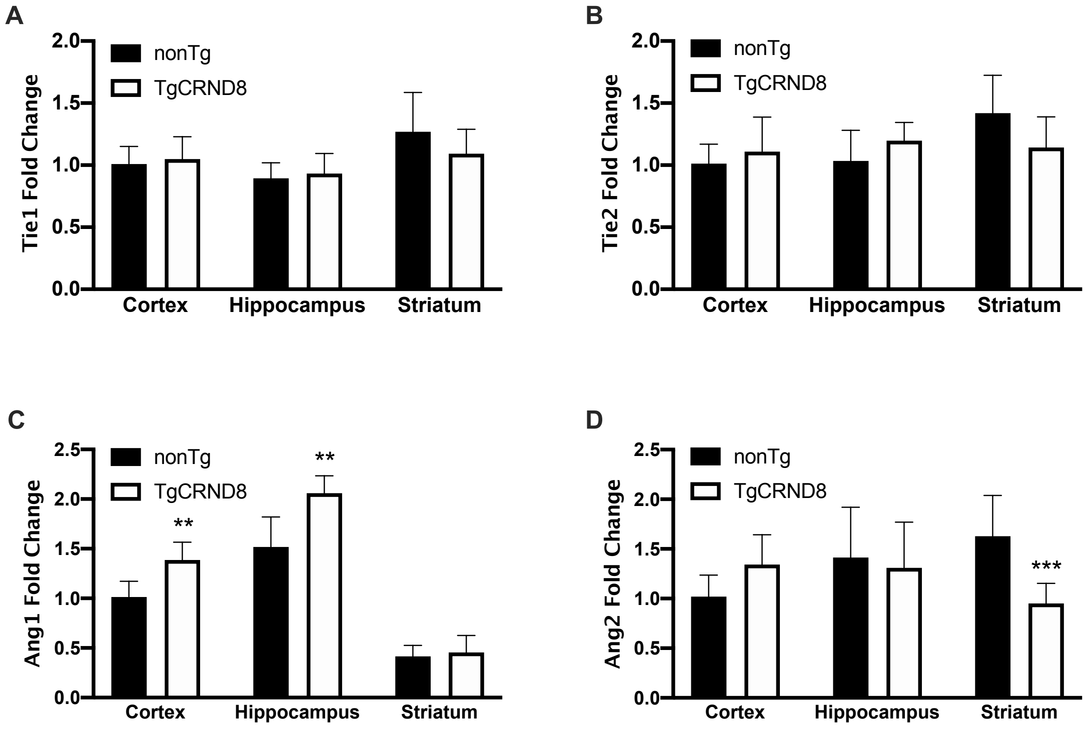

Supp Figure 2: Angpt1 and Angpt2 expression are modulated in TgCRND8 mice. Expression of Tie1 (A), Tie2 (B), Angpt1 (C) and Angpt2 (D) in the cortex, hippocampus and striatum of 7.5-month old TgCRND8 mice. Fold change is relative to the cortex of non-transgenic mice. Analysis done by repeated measures 2-way ANOVA with Sidak's post-hoc test *$p<0.05$, **$p<0.01$, ***$p<0.001$, ****$p<0.0001$.
